# Leflunomide Sustained Skin Delivery Based on Sulfobetaine-Modified Chitosan Nanoparticles Embedded in Biodegradable Polyesters Films

**DOI:** 10.3390/polym13060960

**Published:** 2021-03-21

**Authors:** Stavroula G. Nanaki, Evi Christodoulou, Nikolaos D. Bikiaris, Afroditi Kapourani, Konstantinos N. Kontogiannopoulos, Souzan Vergkizi-Nikolakaki, Panagiotis Barmpalexis

**Affiliations:** 1 Laboratory of Polymer Chemistry and Technology, Department of Chemistry, Aristotle University of Thessaloniki, 54124 Thessaloniki, Greece; sgnanaki@chem.auth.gr (S.G.N.); evicius@gmail.com (E.C.); nbikiaris@gmail.com (N.D.B.); 2 Department of Pharmaceutical Technology, School of Pharmacy, Aristotle University of Thessaloniki, 54124 Thessaloniki, Greece; akapourag@pharm.auth.gr (A.K.); kkontogi@cheng.auth.gr (K.N.K.); 3 Natural Products Research Centre of Excellence-AUTH (NatPro-AUTH), Center for Interdisciplinary Research and Innovation (CIRI-AUTH), 57001 Thessaloniki, Greece; 4 Department of Microbiology, School of Medicine, Faculty of Health Sciences, Aristotle University of Thessaloniki, 54124 Thessaloniki, Greece; sverg@auth.gr

**Keywords:** chitosan, nanoparticles, ionotropic gelation, leflunomide, SDAEM-grafting, mPEG-b-PLA, PLGA, spin coating, biodegradable polyesters, thin-film patches

## Abstract

The aim of the present study was to prepare a leflunomide (LFD) sustained release transdermal delivery system for the treatment of psoriasis. In this context, LFD-loaded nanoparticles (NPs) based on either neat chitosan (CS) or CS modified with [2-(methacryloyloxy)ethyl]dimethyl-(3-sulfopropyl)ammonium hydroxide (SDAEM, a sulfobetaine zwitterionic compound) were initially prepared *via* ionotropic gelation and characterized in terms of in vitro dissolution, physicochemical, and antibacterial properties. Results showed that the use of the SDAEM-modified CS resulted in the formation of LFD-loaded NPs with improved wetting and solubilization properties, better in vitro dissolution profile characteristics (i.e., higher dissolution rate and extent), and improved (enhanced) antibacterial properties. The resultant LFD-loaded NPs were then embedded in suitable thin-film skin patches, prepared *via* spin-coating, utilizing two different biodegradable polyesters, namely methoxy polyethylene glycol-*b-*poly(L-lactide) (mPEG-*b*-PLA, at a ratio of 25/75 mPEG to PLA) and poly(lactic-co-glycolic acid) (PLGA at a ratio of 75/25 DL-lactide/glycolide copolymer). Results showed the formation of polymeric thin-films with no agglomeration (or trapped air) and uniform structure in all cases, while the LFD-loaded NPs were successfully embedded in the polymeric matrix. Analysis of the obtained in vitro dissolution profiles revealed a sustained release profile of the drug for up to approximately twelve days, while between the two proposed systems, the use of CS-SDAEM NPs (independently of the polyester type) was the most promising formulation approach.

## 1. Introduction

Among the several autoimmune diseases reported today, psoriasis is a severe recurrent skin disorder that presents rigorous inflammation [1,2,3], with recent studies showing that at least 5% of the global population suffers from this condition [4]. From an etiological basis, psoriasis is affected by several endogenous or exogenous factors (including heritance, environmental factors, stress, drugs, and alcohol) [5]. Generally, the disorder is characterized by layered inflamed erythematous skin plaques, reinforced by silvery scales [2,6]. Until now, the most commonly employed approach to overcome the symptoms related to psoriasis has included the usage of orally administrated drug medication, especially in cases associated with severe symptoms [7,8]. Among the several drugs used, Leflunomide (LFD) is one of the most commonly administrated *per os* immunomodulator and disease-modifying antirheumatic drug (DMARD) active pharmaceutical ingredients (API) [9,10,11]. Notwithstanding LFD’s good therapeutic outcome, its oral administration is associated with liver toxicity and several gastrointestinal adverse events (such as loose bowel, queasiness, and dyspepsia) [9,12]. Furthermore, the frequent (daily) administration of the oral treatment is associated with problems related to treatment compliance (especially in the case of elderly populations). Based on these limitations, some recent studies (although limited in number) are focusing on the preparation of dermal/transdermal LFD skin delivery formulations, in order to alleviate these side effects [13,14]. However, despite the promising results published, especially by Bae and Park [15], the preparation of LFD-based skin patches possesses several drawbacks related to the physicochemical properties of the API, such as its extremely low aqueous solubility, leading to restrictions in drug skin absorption. Hence, in order to improve the physicochemical properties of LFD and prepare an efficient LFD skin patch, the combination of a modern skin drug delivery strategy, such as the use of chitosan (CS) nanoparticles (NPs) with biodegradable polymeric matrices forming suitable thin-films, seems to be a promising formulation approach. It is important to state that, although according to the National Nanotechnology Initiative, nanotechnology refers to structures roughly in the 1−100 nm size regime, pharmaceutical systems (or even pure drugs) that are up to several hundred nanometers in size (up to ~1000 nm) have been considered as nano-formulations, due to their remarkably different properties (as compared to formulations in micro-scale) and their unique interaction with the human body [16].

CS is a hemi-synthetic cationic linear polysaccharide, synthesized by the deacetylation of chitin [17,18]. It is non-toxic, highly biocompatible, and biodegradable, with low immunogenicity, and as such, it is an excellent candidate for dermal or transdermal pharmaceutical applications [17]. CS and its derivatives have been successfully employed as skin or wound dressing materials under the form of fibers, hydrogels, membranes, scaffolds, or sponges [19,20,21]. In addition to the above-mentioned features, CS exhibits good antibacterial properties, with a diverse inhibition efficiency against different fungi, and Gram-positive and Gram-negative bacteria [22,23]. However, keeping in mind that: (1) psoriatic lesions are characterized by higher bacterial load [24], and that (2) skin patches induce the development of a significant bacterial load as a result of the moisture trapped during their application [25,26], the use of an additional antibacterial agent is mandatory in most cases. For this reason, one promising category of materials that can modify (and enhance) the antibacterial properties of CS are the zwitterions, including the derivatives of carboxybetaine, phosphobetaine, or sulfobetaine [21,27,28,29,30,31,32,33,34,35,36,37,38,39]. More specifically, a recently published study from our group showed that [2-(methacryloyloxy)ethyl]dimethyl-(3-sulfopropyl)ammonium hydroxide (SDAEM)-modified CS may be successfully used to prepare innovative skin emulsions with enhanced antioxidant, antibacterial, and UV protection properties [40], indicating that the CS-SDAEM modification, proposed also herein, may be an extremely promising strategy for the preparation of LFD skin patches.

Several biodegradable polymers (or copolymers) have been proposed as drug delivery matrices for the production of drug-loaded skin patches, including poly(amino acids), poly(orthoesters), poly(alkyl-a-cyano acrylates), poly (acrylamides), and poly(esters) [41,42,43,44]. Among them, poly(lactic-co-glycolic acid) (PLGA) is a GRAS-listed and pharmacopoeia-approved, biodegradable and biocompatible aliphatic polyester, which has shown significant advantages in the preparation of thin-film patches. Additionally, the use of similar polyesters based on diblock PEG- and PLA-based copolymers, such as methoxy PEG-*b*-poly(L-lactide) (mPEG-b-PLA), may also represent a promising approach, since it seems to improve the limitations associated with PLGA, such as the high hydrophobicity, the entrapment by macrophages through the opsonization process, the long-term degradation time, and the formation of an *in situ* acidic microenvironment [45,46,47].

Therefore, the aim of the present study was to evaluate, for the first time, the use of SDAEM-modified CS as a suitable NP drug encapsulation carrier in the preparation of polyester-based LFD skin patches, appropriate for the treatment of psoriasis. For this reason, SDAEM-modified CS was initially synthesized by free radical polymerization and used for the encapsulation of LFD in NPs prepared by ionotropic gelation. After the full characterization of the prepared NPs, mPEG-b-PLA 25/75 (synthesized *via* ring opening polymerization) and PLGA 75/25 LFD-loaded thin-films were prepared, using a spin-coating technique, and thoroughly evaluated.

## 2. Materials and Methods

### 2.1. Materials

**Figure 1:**
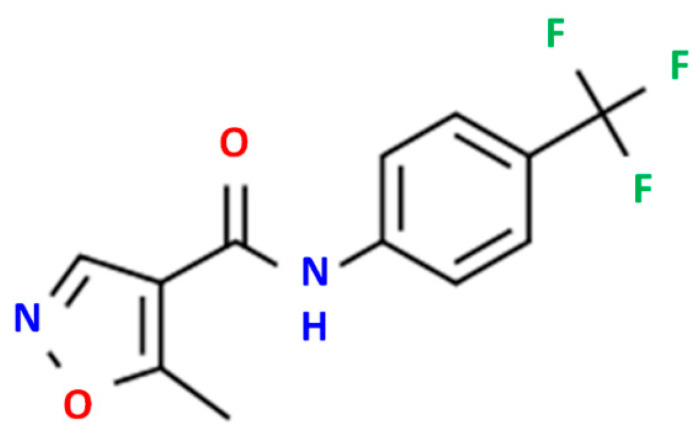
LDF chemical structure.

High-purity LFD (Figure 1) was kindly donated by Pharmathen S.A. (Athens, Greece). CS of low molecular weight (50–190 kDa, degree of deacetylation 75–85%) and sodium tripolyphosphate (TPP), used as ionic crosslinker, were purchased from Sigma-Aldrich Co. (Steinheim, Germany). [2-(methacryloyloxy)ethyl]dimethyl-(3-sulfopropyl)ammonium hydroxide (SDAEM) with 95% purity was purchased from Fluorochem (London, UK), while potassium persulfate (K_2_S_2_O_8_, KPS, purity 99.0%) was purchased from Merck (Athens, Greece). L-lactide (98+%) (S,S)-3,6-Dimethyl-1,4-dioxane-2,5-dione was purchased from Alfa Aesar Chemicals (Kandel, Germany). Methoxy poly(ethylene glycol) (average Mw 2000), tin(II) 2-ethylhexanoate (TEH) (96%) catalysts, and poly(vinyl alcohol) (PVA, Mw 13,000–23,000, 87–89% hydrolyzed) were all obtained from Sigma-Aldrich chemical company (Saint Louis, MO, USA). PLGA 75/25 (PURASORB^®^ PDLG 7507) was purchased from Corbion (Gorinchem, The Netherlands). All other solvents or reagents used were of analytical grade and purchased also from Sigma-Aldrich.

### 2.2. Synthesis of SDAEM-Modified CS

**Scheme 1:**
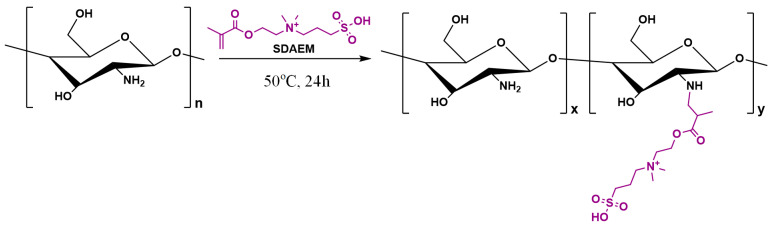
Chemical reaction followed during the synthesis of SDAEM-modified CS.

Synthesis of SDAEM-modified CS (CS-SDAEM) was carried out *via* free-radical polymerization, based on previous reports [40,48]. Briefly, 10 g of CS were dissolved in 400 mL of aqueous acetic acid solution at 2% *v/v* concentration. The mixture was left under magnetic stirring at room temperature. After complete chitosan dissolution, 0.6 g of SDAEM and 4.8 mg of KPS were added into the mixture. The grafting reaction took place at 50 °C, for 24 h, under nitrogen atmosphere and continuous magnetic stirring, leading to the formation of a viscous liquid. The product was then precipitated by an aqueous solution of NaOH (1 M in concentration). The resulting precipitate was collected, frozen, and freeze-dried at −56 °C. The final product was further purified by Soxhlet using methanol as an eluent, in order to remove any unreacted SDAEM monomer. The structures and the reaction conditions are shown in the following Scheme 1:

### 2.3. Characterization of SDAEM-Modified CS

#### 2.3.1. Nuclear Magnetic Resonance (^1^H-NMR)

^1^H-NMR spectra of samples were obtained using an Agilent spectrometer (Agilent Technologies, Santa Clara, CA, USA) operating at a frequency of 500 MHz at RT. The samples were dissolved in deuterated acetic acid at 2% *v/v* solution in deuterated water. The spectra were internally referenced with tetramethylsilane (TMS) and calibrated using the residual solvent peak. The number of scans was 16, and the sweep width was 6 kHz.

#### 2.3.2. FT-IR

FT-IR spectra of samples were obtained using an FT-IR spectrometer (model FTIR-2000, Perkin Elmer, Waltham, MA, USA). A small amount of each sample was triturated with a proper amount of potassium bromide (KBr), and disks were formed under pressure. The spectra were collected in the range of 400 to 4000 cm^−1^, at a resolution of 4 cm^−1^, using 64 co-added scans, and the baseline was corrected and converted into absorbance mode.

#### 2.3.3. pXRD

X-ray powder diffraction (pXRD) patterns were recorded using an XRD-diffractometer (Rigaku-Miniflex II, Chalgrove, Oxford, UK) with a CuKα radiation for crystalline phase identification (λ = 0.15405 nm). The samples were scanned over the 2*θ* range of 5 to 55°, with a scan speed of 1°/min.

#### 2.3.4. Swelling Study

Swelling ability was evaluated by measuring the water sorption capacity in water (pH = 7.4). Briefly, CS or CS-SDAEM samples, after being carefully weighed (W_0_), were inserted in water for several hours. At predetermined time intervals, the samples were removed, wiped off by filter paper in order to remove the excess surface water, and weighed in order to determine the swelling weight (W_n_). The percentage weight increase of samples during the swelling experiment (i.e., degree of swelling) was calculated by the following equation:

Degree of Swelling (%) = [(W_n_ − W_0_)/W_0_] × 100 (1)

### 2.4. Preparation of NPs

LFD-loaded CS (or CS-SDAEM) NPs were prepared according to a previously published modified ionotropic gelation method, using TPP as ionic crosslinker and PVA as solubility enhancer [40]. Briefly, 300 mg of neat CS (or CS-SDAEM) was dissolved in 20 mL acetic acid aqueous solution (1% *v/v* in concentration). Then, 20 mL of an aqueous solution of PVA (1% *w/v*) was added into the CS (or CS-SDAEM) solution, and 20 mg of LFD (dissolved in 5 mL dichloromethane, DCM) was inserted into the final solution. Probe sonication was conducted for 1 min, which resulted in the formation of an oil-in-water emulsion (o/w). Afterwards, 37.5 mL of aqueous solution of TPP (2 mg/mL) was added dropwise, and the resultant nanosuspension was left under mild stirring for 24 h. Nanoparticles were collected by centrifugation (19,216 rcf for 20 min). The obtained nanoparticles were washed once with water and the obtained aqueous nano-suspension was lyophilized using a Scanvac freeze-drier system (Coolsafe 110-4 Pro, Labogen Scandinavia) for 24 h at −56 °C, in order to obtain the final dried NPs.

### 2.5. Characterization of NPs

#### 2.5.1. Yield, Drug-Loading, and Encapsulation Efficiency (EE)

The determination of drug loading and EE was performed by dispersing 10 mg of the prepared nanoparticles in 10 mL of DCM. The resulting suspension was stirred for 24 h and filtered using PTFE hydrophobic filters of 0.45 nm pore size. The LFD content was determined using a Shimadzu HPLC prominence system (Kyoto, Japan), consisting of a degasser (DGU-20A5, Kyoto, Japan), a liquid chromatograph (LC-20 AD, Kyoto, Japan), an autosampler (SIL-20AC, Kyoto, Japan), a UV/Vis detector (SPD-20A, Kyoto, Japan), and a column oven (CTO-20AC, Kyoto, Japan). A CNW Technologies Athena C18, 120 A, 5 μm, 250 mm × 4.6 mm analytical column was used, and the analysis was performed at 25 °C. The mobile phase consisted of acetonitrile/water (acidified with phosphoric acid at final pH = 3.0) 80/20 *v/v*, at a flow rate of 1.0 mL/min. UV detection was performed at 295 nm. The injection volume was 20 μL. The calibration curve was created by diluting a stock methanol solution of 500 ppm LFD to concentrations of 0.01, 0.025, 0.05, 0.1, 0.25, 0.5, 1.0, 2.5, 5.0, 7.5, 10.0, 25.0, and 50.0 ppm.

NP’s yield, drug loading, and EE were calculated based on the following equations:

Yield (%) = [Weight of NPs/Initial weight of polymer and drug] × 100,(2)

Drug loading (%) = [Weight of drug in NPs/Weight of NPs] × 100,(3)

EE (%) = [Weight of drug in NPs/Initial weight of drug] × 100(4)

#### 2.5.2. FTIR and pXRD

The FTIR and pXRD analysis of the prepared NPs were performed according to the methodology and equipment described previously.

#### 2.5.3. Differential Scanning Calorimetry (DSC)

For DSC analysis, a PerkinElmer Pyris 1 differential scanning calorimeter (Waltham, MA, USA), calibrated with indium and zinc standards, was used. About 5.0 ± 0.1 mg of each sample was weighed, placed in sealed aluminum pans, and heated up from 20 to 240 °C, with a heating rate of 20 °C/min in an inert atmosphere (N_2_, flow rate 50 mL/min), held in for 3 min, in order to erase any thermal history, cooled to approximately 20 °C with a cooling rate of 20 °C/min, and heated again up to 240 °C, with a heating rate of 10 °C/min.

#### 2.5.4. Particle Size and ζ-Potential

The particle size distribution and ζ-potential of the prepared NPs were determined by dynamic light scattering (DLS), utilizing a Zetasizer Nano Instrument (Malvern Instruments, Nano ZS, ZEN3600, Malvern, UK) equipped with a 532 nm laser, using angle measurements of 90° at 25 °C. The samples were measured in suspension form, using an aqueous solution of NaCl (10^−4^ M) after sonication at 25 °C. For all samples, experiments were performed in triplicate and results are presented in mean values.

Additionally, the particle size and the morphology of the prepared NPs were evaluated *via* scanning electron microscopy (SEM). Specifically, the prepared samples were covered with a carbon coating to provide a good conductivity of the electron beam before examining in a JEOL (JMS-840A) scanning microscope (Jeol Ltd., Akishima, Japan). SEM was performed with an accelerating voltage of 20 kV, probe current of 45 nA, and counting time of 60 s.

#### 2.5.5. In Vitro Dissolution Studies

The in vitro release studies were conducted in a DISTEK Dissolution Apparatus II (North Brunswick, NJ, USA), equipped with an autosampler. Dissolution was performed at 37 ± 0.5 °C, and the rotation speed was set at 50 rpm. The dissolution medium was 500 mL of simulated body fluid (SBF) at pH = 7.4, with 0.1% *v/v* of Tween 20 (used to maintain perfect sink conditions). Two milliliters of aqueous solution were withdrawn from the release media at predefined time intervals and quantified *via* the HPLC method described previously.

#### 2.5.6. In Vitro Antibacterial Activity Testing

The antibacterial activity evaluation of CS and modified CS materials was conducted following the method described previously [49]. Briefly, bacteria suspended in phosphate buffer saline PH 5.8 were exposed to CS and corresponding modified materials for 4 h, and the number of remaining viable cells were estimated by counting the colony-forming units on blood agar plates after an o/n culture. Two bacterial cultures were used for this purpose: *Escherichia coli* (*E. coli*.) for Gram negative, and *Staphylococcus aureus* (*S. aureus*) for Gram positive. Both were well-characterized clinical isolates that were tested biochemically for taxonomical identification and checked for antibiotic resistance. *E. coli* was used at a starting concentration of 1.5 × 10^8^ cfu/mL, and *S. aureus* at 1.8 × 10^8^ cfu/mL, according to 0.5 McFarland. Each tested material was added to 1 mL of each bacterial culture at a concentration of 0.4% *w/v*.

### 2.6. Preparation of mPEG-b-PLA

**Scheme 2:**
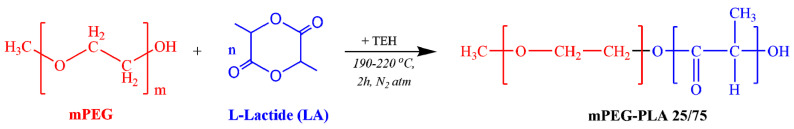
Chemical reaction followed during the synthesis of mPEG-b-PLA.

The mPEG-b-PLA copolymer (in 25/75 mass ratio) was synthesized by ring opening polymerization of L-lactide (Scheme 2).

Before mixing, L-lactide was dehydrated by freeze-drying for 24 h, and then placed in a round-bottom flask charged with the proper amount of mPEG and TEH (used as catalyst at 400 ppm based on the lactide content). The reaction mixture was degassed and, after purging with nitrogen several times, was inserted into a heated salt bath at 190 oc. The reaction was carried out under constant mechanical stirring (250 rpm) and N_2_ atmosphere, while gradually raising the reaction temperature up to 220 °C, over a period of 2 h. To further increase the molecular weight of the produced copolymer and remove the non-reacted monomers, the system was heated to a temperature of 240 oc under increased mechanical stirring and high vacuum (∼5.0 Pa), which was applied slowly, over a period of about 15 min. The reaction flask was then quenched to room temperature (RT), and the products were purified by dissolving them in chloroform. The copolymer was precipitated in cold methanol, twice filtered, and dried in a vacuum oven for 24 h, before being placed in hermetically sealed vails.

### 2.7. Characterization of mPEG-b-PLA

mPEG-b-PLA was fully characterized *via* FT-IR, DSC, and pXRD, following the same methodology and equipment described previously.

^1^H-NMR spectra of samples were obtained using an Agilent spectrometer (Agilent Technologies, Santa Clara, CA, USA), operating at a frequency of 500 MHz at RT. The samples were dissolved in deuterated chloroform (CDCl_3_) at 5% *w/v*. The spectra were in-ternally referenced with tetramethylsilane (TMS) and calibrated, using the residual solvent peak. The number of scans was 16, and the sweep width was 6 kHz.

Additionally, the resultant block-copolymer was evaluated *via* thermogravimetric analysis (TGA). Briefly, TGA was carried out with a SETARAM SETSYS 16/18 TG-DTA (Setaram instrumentation, Lyon, France). Samples (5.0 ± 0.2 mg) were placed in alumina crucibles. An empty alumina crucible was used as reference. Samples were heated from ambient temperature to 600 °C, in a 50 mL/min flow of N_2,_ at a heating rate of 20 °C/min, and the change in sample’s weight was recorded with temperature, in order to obtain the thermal stability profile of the polymer.

In addition to TGA, the polyester was characterized in terms of intrinsic viscosity [η] at 25 °C, using an Ubbelohde viscometer. Specifically, the polymer was dissolved in chloroform (at a concentration of 1% *w/v*) and filtered through a disposable membrane filter (0.2 mm, Teflon) before the measurement. Intrinsic viscosity was calculated using the Solomon–Ciuta equation [50]:

[η] = ([2(t/t_0_ − ln(t/t_0_) − 1)]^1/2^)/c (5)

where c is the concentration of the solution, t is the flow time of solution, and t_0_ represents the flow time of pure solvent.

### 2.8. Preparation and Characterization of LFD Thin-Film Patches

Nanocomposite thin-films, consisting of CS (or CS-SDAEM) NPs embedded in a mPEG-PLA 25/75 (or PLGA 75/25) matrix, were fabricated using the spin-coating method. In brief, each polymer was initially dissolved in a proper amount of dichloromethane, followed by the addition of LFD-loaded chitosan NPs in a concentration of 20% (*w/v*) with respect to the volume of polymer solution. The polymer dispersions were mechanically stirred until complete homogenization. A fixed volume of the polymer mixture was then deposited at the center of a pre-cleaned (by sonication in Milli-Q/ethanol solvent) glass substrate held by vacuum upon the spin-coater disk (Laurell WS-650-23, Laurell Technologies Corporation, North Wales, USA), operating at an angular velocity of 4000 rpm. At the end of the spin-coating process, the nanocomposite thin-films were dried overnight in a vacuum oven at room temperature, before storage in hermetically sealed vials. All films were prepared in triplicates.

The prepared thin-films were characterized in terms of FT-IR, DSC, and pXRD, using the methodology and equipment described in the previous sections. Additionally, in vitro dissolution studies were performed using a DISTEK Dissolution Apparatus II (North Brunswick, NJ, USA), equipped with an autosampler. The thin-films were held in appropriate transdermal patch holders, which were purchased from QLA Company (USA). Dissolution was performed at 37 ± 0.5 °C and 50 rpm. The dissolution medium was 500 mL of simulated body fluid (SBF), adjusted at pH = 7.4 with 0.1% *v/v* of Tween 20. Two milliliters of aqueous solution were withdrawn from the release media at predefined time intervals and analyzed for LFD content *via* the HPLC method described previously.

Additionally, swelling ratio (degree of swelling) was measured in SBF. Each patch was carefully weighed (W_1_) and immersed in SBF at 37 °C. The remnants of materials were wiped of excess surface water using filter paper and weighed (W_2_) at different time intervals until constant weight. The swelling ratio was calculated at different time intervals using the following equation:

Swelling ratio = (W_2_ − W_1_)/W_1_ × 100 (6)

## 3. Results and Discussion

### 3.1. Characterization of CS-SDAEM Grafted Material

As stated earlier, CS modification with SDAEM was achieved *via* free-radical polymerization, using a low SDAEM-to-CS concentration. This low SDAEM content (lower than previous attempts) was used in order to retain a good portion of CS free amino groups during polymerization (a necessary feature in order to maintain the good CS mucoadhesive properties) and harmonize with modern pharmaceutical formulation strategies, calling for the use of low antibacterial/antimicrobial quantities.

**Figure 2:**
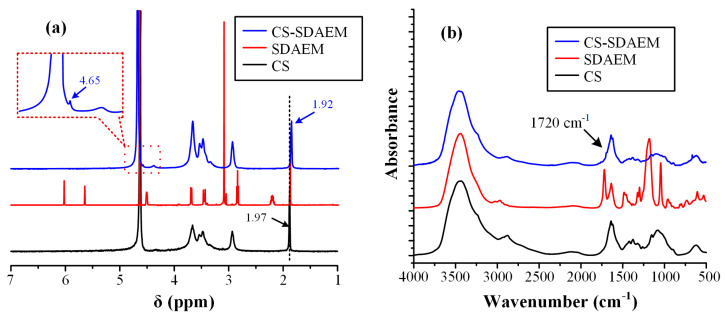
^1^H-NMR spectra (**a**) and FT-IR spectra (**b**) of CS, SDAEM and CS-SDAEM.

In order to evaluate and prove the successful modification of CS with SDAEM, nuclear magnetic resonance spectroscopy (^1^H-NMR) was initially used. Figure 2a shows the ^1^H-NMR spectra of the neat CS, the neat SDAEM, and the modified SDAEM-CS, respectively.

In respect to the neat CS, the ^1^H-NMR peak recorded at 1.97 ppm can be attributed to the methyl protons of the N-acetyl group of the polysaccharide, while the peaks between 2.50 and 5.00 ppm arise from the protons of the glucosamine unit. Looking at the ^1^H-NMR spectra of the SDAEM-modified CS, the peak of neat CS, located initially at 1.97 ppm, is now shifted to a slightly lower ppm (i.e., at 1.92 ppm), a clear indication that the SDAEM monomer was successfully embedded in the CS backbone. In addition, a new small peak recorded at 4.65 ppm is indicative of the methyl group, which is located next to the oxygen of the ester group of the monomer. Finally, as in previous studies using higher SDAEM concentrations [40], the absence of the SDAEM spectrum peaks at 5.80 and 6.18 ppm (corresponding to the double bonds of the monomer) is indicative of the reaction evolving between the two components (CS and SDAEM); it also provides strong evidence that all monomers have either reacted with CS or that the Soxhlet extraction purification method employed was adequate enough to remove all un-reacting SDAEM monomers.

In addition, the grafting reaction of CS with SDAEM was evaluated also *via* FT-IR (Figure 2b). In the case of pure CS, results showed several characteristic peaks. Specifically, a broad peak was recorded at 3458 cm^−1^ (due to the hydroxyl groups vibrations), two shoulders were recorded at 3259 and 3088 cm^−1^ (attributed to the primary and secondary amino groups of CS, respectively), two peaks at 1659 and 1592 cm^−1^ (due to the absorption amide I and II, respectively), a peak at 1381 cm^−1^ (attributed to the –CH_2_ bending), a peak at 1153 cm^−1^ (due to the asymmetric stretching of the C–O–C bridge), and finally, two peaks at 1075 and 1033 cm^−1^ (attributed to the skeletal stretching vibration of CO). In the case of neat SDAEM, results showed a peak at 1722 cm^−1^ (due to the C=O stretching vibrations), a small shoulder-peak at 1636 cm^−1^ (due to the stretching vibrations of the =CH vinyl group), a peak at 1293 cm^−1^ (attributed to the C–N vibrations), and two peaks at 1039 and 1187 cm^−1^ (attributed to the symmetrical and asymmetrical stretching vibrations of S=O bond, respectively). Additionally, in the case of CS-SDAEM, a small shoulder was recorded at 3431 cm^−1^ (attributed to the -OH vibrations of CS), two peaks at 3357 and 3294 cm^−1^ (attributed to hydrogen bonded –OH groups and primary amino groups, respectively), a small shoulder at 3084 cm^−1^ (due to the secondary amino groups), a small peak at 1720 cm^−1^ and another at 1650 cm^−1^ (due to the ester groups of CS), a small peak at 1200 cm^−1^ (attributed to the SO_3_^−^ group vibrations), and a strong peak at 1027 cm^−1^ (attributed to the C–N absorption vibrations). These peaks, and especially the new peak at 1720 cm^−1^ corresponding to the ester group of the monomer, which is in agreement with previous results [40,51], indicate the successful grafting of CS and the formation of a CS-SDAEM, as well as the formation of intermolecular interactions (probably hydrogen bonds, HBs) between the reactive groups of the SDAEM monomer (–CO–O and SO_3_^–^) and the -NH_2_ or the –OH groups of CS.

**Figure 3:**
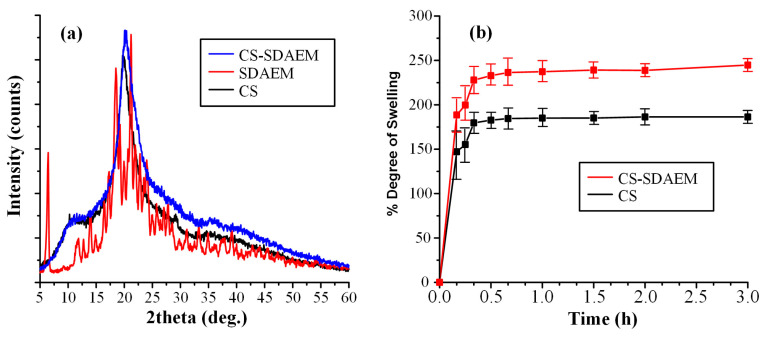
pXRD diffractograms (**a**) and swelling profile (**b**) of CS, SDAEM, and CS- SDAEM.

Following the above analyses and the verification of the CS-SDAEM synthesis, the physical state of the new derived material was evaluated *via* pXRD (Figure 3a).

In regard to the neat CS, the obtained diffractogram showed that the polymeric material is semicrystalline with a characteristic amorphous halo (recorded from 10 to 40 deg) and two diffractogram peaks at 11.0 and 21.0 deg, respectively. On the contrary, neat SDAEM is a crystalline material with several characteristic peaks recorded at 2θ of 6.4, 18.5, and 21.3 deg. In regard to the newly derived CS-SDAEM, results showed that this is also semi-crystalline in nature, with a characteristic amorphous halo and a single broad pXRD peak at 20.1 deg. It should be noted that, compared to previous results utilizing SDAEM at higher concentrations [40,51], the resultant CS-SDAEM polymer in the present study showed a similar degree of crystallinity as compared to the neat CS, indicating that at such low concentrations, SDAEM is not adequate enough to reduce CS’s ability to fold and create crystallites.

In the final step of CS-SDAEM evaluation, the swelling ability of the newly grafted polymer was evaluated and compared to the neat CS. In general, previous studies have shown that wettability (expressed by swelling ability) is one of the most important prerequisites for a successful and prolonged adhesion of a material on the skin (or the mucus) surface. Higher water uptake leads to the formation of a more stable gel, which, subsequently, leads to the formation of stronger interactions between the CS and the skin (or mucus) surface. In this set framework, Figure 3b shows the degree of swelling (at pH 7.4) for the neat CS and the CS-SDAEM derivative. Results showed that CS reached its maximum swelling degree at ~175% in the first 30 min, with no further changes for up to 3 h. Similarly, the SDAEM-modified CS showed the same swelling behavior, although a slightly higher degree of swelling was achieved (~250% in 3h). These findings indicate that the newly prepared polymer is slightly more hydrophilic than the neat CS, a feature attributed to the additional reactive groups of SDAEM embedded in the CS backbone chain. This finding is in agreement with swelling results reported previously; however, it is important to note that compared to those reports, the utilization of higher amounts of SDAEM [40,51] leads to a significantly lower enhancement of CS’s swelling ability, as the results in the present study show. This reduction in hydrophilicity may compromise the material’s skin adhesion ability; however, in contrast to the previous reports, the obtained drug-loaded NPs, resulting from the SDAEM-modified CS, will be embedded into a thin-film patch prepared by aliphatic polyesters (mPEG-b-PLA or PLGA), which have excellent skin adhesive properties on their own, and hence it is anticipated that the reduction in adhesion coming from the low SDAEM concentration will be compensated by the presence of the polyester matrix.

### 3.2. LFD-Loaded NPs

The main scope of the present study was to prepare a suitable LFD skin patch for the treatment of psoriasis. In order, however, to succeed at this, the poor aqueous solubility of LFD should be improved. In this vein, the present study evaluates, for the first time, the use of CS-SDAEM NPs for enhancing a drug’s wetting characteristics and solubilization, while simultaneously improving the inherent CS antibacterial properties.

In the present study, LFD-loaded NPs (with neat or modified CS) were prepared *via* the ionic gelation method, since this is a simple technique capable of spontaneously forming NPs through mild conditions. Following this technique, CS NPs with good particle size (i.e., below 1000 nm) were prepared in all cases (Table 1). It should be noted that (as stated in the introduction), although nanotechnology refers to structures having NP size up to 100 nm (at least in one dimension), in pharmaceutical applications it is common to use the term in formulations (or even pure APIs) having particle size up to several hundred nanometers, due to their remarkably different properties (as compared to micro-scale formulations) and their unique interactions with the human body [16].

Looking closer to the obtained results, it seems that the use of modified CS-SDAEM results in the formation of larger NPs as compared to neat, unmodified CS. This finding contradicts previous reports in which the presence of SDAEM has led to NPs’ size reduction [40], while other studies have shown similar increasing size effects [51]. Hence, based on these results, it seems that the quantity of SDAEM used during the CS grafting process, as well as the several ionic gelation process parameters, play a significant role in determining the final NP particle size. This is more easily understood keeping in mind that SDAEM contains both positive and negative charges, which, depending on the compound’s final concentration and grafting backbone position, may significantly affect the ionic interactions evolving during the ionic gelation process. Returning to the results reported in Table 1, incorporation of the API into the prepared systems showed a further increase of NPs’ size. However, despite this increase, all prepared NPs had acceptable particle size (less than 800 nm), while among the prepared systems CS-SDAEM NPs showed adequate polydispersity as compared to the rest formulations (although some multimodality was observed).

**Figure 4:**
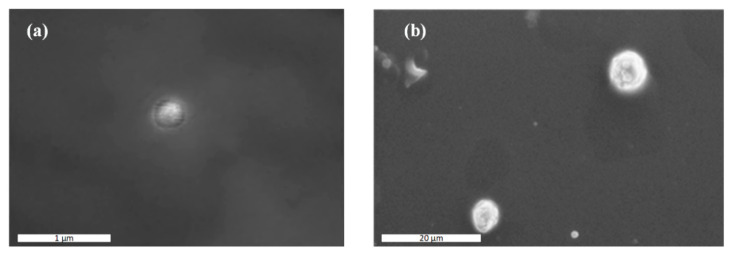
SEM images of CS-LFD (**a**) and CS-SDAEM-LFD (**b**) NPs.

In an attempt to further evaluate the actual NPs’ size and take a closer look at the morphology of the prepared NPs, SEM analysis was performed. Images presented in Figure 4 show that the actual size of the CS-LFD NPs was ~300 nm, while CS-SDAEM-LFD NPs’ size was defined as equal to ~700 nm in size. In addition, results showed that the prepared NPs had good spherical shape and, in the case of CS-SDAEM-LFD NPs, a rather abrasive surface.

In addition to particle size, ζ-potential of the prepared NPs was also evaluated. It should be noted that ζ-potential presents an indirect measure of NPs’ stability profile, with values between −10 mV and +10 mV showing rapid agglomeration, leading to serious long-term stability problems. In general, looking at previously published results, CS NPs prepared *via* the ionic gelation show high positive ζ-potential values [52]. This is also true in the present study, where positive ζ-potential values were recorded for all prepared CS NPs (Table 1). Specifically, in the case of neat CS NPs, a ζ-potential value of +59.9 mV was recorded, which was reduced at +39.5 mV with the addition of SDAEM in the backbone structure of the polymer. Similar results were obtained in the case of drug-loaded NPs with LFD-CS NPs, showing ζ-potential value of +57.7 mV, as compared to +40.0 mV obtained for CS-SDAEM NPs. However, despite the small drop in the ζ-potential values of the SDAEM-grafted CS NPs observed, irrespectively of LFD presence, all obtained ζ-potential values were well above the +10 mV, indicating good NP stability.

Finally, in regard to drug-loading and EE, results in Table 1 show that the use of SDAEM-modified CS resulted in slightly lower drug-loadings and EE values. This can be attributed to the presence of steric hindrance phenomena, induced by SDAEM. However, despite this difference, similar yields were achieved in all cases.

**Figure 5:**
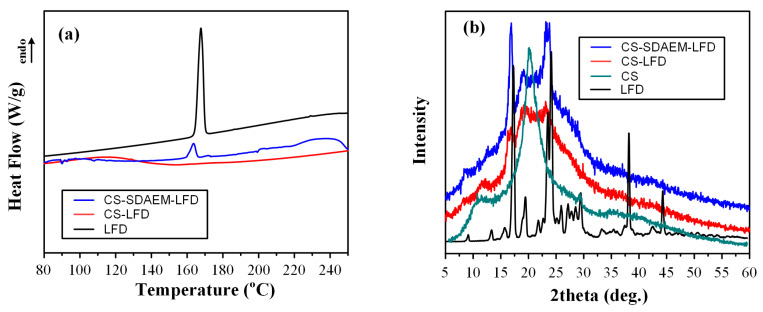
DSC thermograms (**a**) and pXRD diffractograms (**b**) of LFD loaded CS and CS-SDAEM NPs.

Moving to the physical state characterization, the obtained NPs were initially evaluated using DSC (Figure 5a).

Results showed that the neat API is a crystalline drug, with a characteristic melting endotherm located at 167.7 °C. Interestingly, when the API was encapsulated into the neat CS NPs, it was amorphously dispersed (no LFD endothermic peaks were present in the obtained thermogram), while the use of SDAEM-modified CS showed that a portion of the API remained crystalline (a small melting endotherm was recorded). This probably indicates that the SDAEM steric hindrance phenomena evolving in the backbone chain of the CS leads to the formation of API dimers, which in turn leads to the drug’s nucleation and recrystallization. Based on this finding, we may assume that LFD forms stronger intermolecular interactions with the unmodified CS (inhibiting thus the formation of LFD dimers), as compared to the modified SDAEM-CS (this hypothesis will be further evaluated in the following FT-IR analysis section). What is also important to note is that in the case of LFD-loaded SDAEM-CS NPs, the obtained API melting endotherm is recoded in a lower temperature as compared to the neat API (163.5 °C compared to 167.7 °C), indicating that some kind of intermolecular interactions are taking place between the API and the NP’s matrix, although this difference may be attributed to the melting point depression of the API that is present in the mixture.

In a further step, and in order to confirm the DSC suggested physical state changes, pXRD diffractograms were also recorded. Results in Figure 5b showed that the neat LFD is a high-crystalline API with characteristic diffractogram peaks at 2θ of 17.0, 19.6, 23.6, 24.2, 38.4, and 44.6 deg. In the case of drug-loaded NPs, results showed a clear reduction in CS’s crystallinity, regardless of the type of CS used (i.e., grafted or neat CS). This is a typical finding reported also in previous studies using ionic gelation with TPP for the preparation of CS NPs, and is attributed to the rearrangement of the intermolecular and intramolecular network of the CS polymer, resulting from the crosslinking reaction with the TPP ions. In regard to the physical state of the API encapsulated in the prepared NPs, the obtained diffractograms showed the presence of some major LFD pXRD peaks at 2θ of 17.0 and 23.6 deg, indicating that in contrast to the DSC suggestions, pXRD reveals that the API remained crystalline regardless of CS modification. However, it is important to note that the use of neat, unmodified CS resulted in the reduction of API’s crystallinity as compared to SDAEM-CS, probably indicating that stronger molecular interactions are being formed between the API and the unmodified CS.

**Figure 6:**
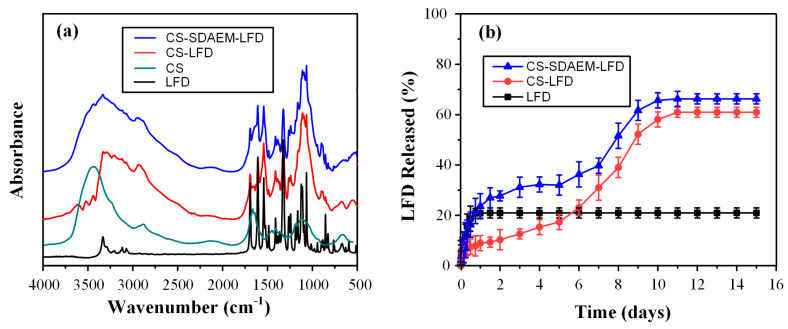
FT-IR spectra (**a**) and in vitro dissolution profile (**b**) of LFD loaded CS and CS-SDAEM NPs.

Following the above physical state analysis, the formation of LFD-CS molecular interaction was evaluated *via* FT-IR. Figure 6a shows the FT-IR spectra of the neat LFD as well as the spectra of the obtained NPs.

Regarding LFD, results showed the API characteristic FT-IR peaks at 3333 cm^−1^ (attributed to NH peak of amide), 2924 cm^−1^ (assigned to CH stretching vibration), 1690 cm^−1^ (attributed to the HC=N–O of the isoxazole ring), 1604 cm^−1^ and 1540 cm^−1^ (assigned to the C=O vibrations of the amide group), and 1504 cm^−1^ (attributed to C=C stretching vibrations). In comparison with LFD-loaded, CS NPs showed a significant shift in the peaks recorded in the regions of 3540 to 3290 cm^−1^ and 1660 to 1550 cm^−1^, indicating the formation of molecular interactions (probably HBs) between the amino and the hydroxyl groups of CS with the carboxamide or the trifluoromethylphenyl groups of the API. Similar shifts, although to a lesser extent, were also observed in the FT-IR spectrum of the LFD-loaded NPs using SDAEM-CS instead of neat CS, indicating that weaker interactions take place between the API and the modified CS polymeric chain. This verifies the previous hypothesis, suggesting that the reduced API crystallinity in the case of CS NPs is due to the formation of stronger molecular interactions between the API and the polymeric matrix.

Drug in vitro dissolution profiles are presented in Figure 6b. Regarding the pure API, results showed that ~20% of LFD was dissolved within the 1st day of dissolution with no further changes for up to 15 days, a result indicative of API’s extremely low aqueous solubility. On the contrary, drug-loaded CS nanoparticles showed an initial burst release in the first 12 h (~10% of API was released), followed by a biphasic release with an initial zero-order release profile (i.e., constant release rate) for up to almost 6 days (~20% of the API was released), followed by a first order (Fickian) release profile for up to approximately 11 days, where almost the 60% of the API was released. Hence, it is clear from the obtained dissolution results that the encapsulation of the API within the CS nanoparticles resulted in a tremendous enhancement of API’s solubilization, which is a crucial feature in order to prepare a successful LFD skin patch. This improvement can be attributed partially to the increased wetting properties of the CS-based NPs and to the partial amorphization of the API within the prepared nanostructure, although it is most likely that the formed nano LFD crystals within the CS NPs also exhibit a substantially higher dissolution and solubilization rate compared to the pure API micro-scale crystals. In the case of the SDAEM-CS modified NPs, a similar biphasic release profile was observed, although different release mechanisms were recorded. Specifically, in the case of SDAEM-CS NPs, an initial first order release profile was observed up to 4 days (where ~30% of the API was released from the NPs), followed by a sigmoid release profile for up to ~10 days, where almost 70% of the API was released from the drug-loaded NPs. These differences can be attributed to the improved wetting (and swelling) properties of the SDAEM monomer embedded in the CS backbone chain. Comparison of the two NPs’ formulations showed that the modification of CS with SDAEM was able to further improve the API’s solubilization and dissolution rate, and hence it can be assumed to be a better carrier for the encapsulation of LFD and the preparation of LFD thin-film skin patches.

In the final step of the LFD-loaded NPs’ characterization, the antibacterial properties of the prepared NPs were evaluated, and the results are presented in Table 2.

The results show that the neat CS has some antibacterial properties (*p*-value < 0.05) against both bacteria, as compared to the neat API, where it was negligible. As hypothesized initially, the CS modification with SDAEM resulted in the formation of NPs with increased antibacterial properties (again with *p*-value < 0.05), a feature that can be attributed to the presence of the quaternary ammonium groups in the SDAEM monomer. It should be noted that compared to previous studies evaluating the use of CS-SDAEM NPs in pharmaceutical skin formulations [40], the present study has shown that the use of extremely low SDAEM concentrations is still adequate in order to enhance the antibacterial properties of the resultant modified NPs.

Hence, based on the above findings, it is obvious that the use of SDAEM and the preparation of a new SDAEM-CS grafting polymer resulted in the formation of LFD-loaded NPs having better dissolution profile characteristics (higher dissolution rate and extent) and improved antibacterial properties, compared to NPs prepared with unmodified CS.

### 3.3. Synthesis and Characterization of mPEG-b-PLA

As stated in the introductory section, the developed LFD skin patches will be based on the preparation of thin-films, using matrices of two different types of aliphatic polyesters: (1) mPEG-b-PLA and (2) PLGA. Out of them, the former (i.e., mPEG-b-PLA) was synthesized in the present study *via* ring-opening polymerization of L-lactide. The successful synthesis of the resultant block-copolymer was evaluated *via* ^1^H NMR and FT-IR, while its thermal properties and physical state characteristics were evaluated *via* DSC, TGA, and pXRD analysis.

**Figure 7:**
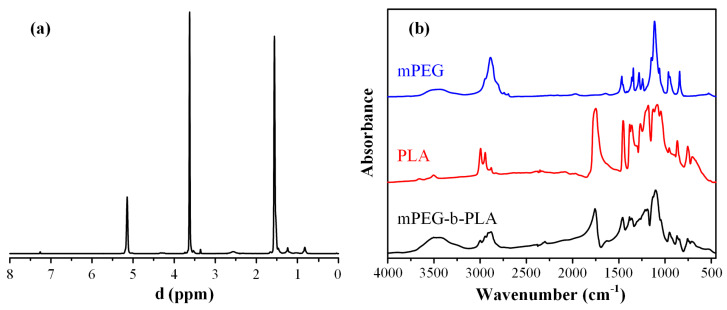
^1^H-NMR (**a**) and FT-IR spectra (**b**) of mPEG-b-PLA and monomers.

Figure 7a shows the obtained ^1^H-NMR spectrum for the newly synthesized block copolymer. Based on the obtained results, the peaks at 1.56 and 5.14 ppm belong to a methyl (–CH_3_) and methine proton (–CH) of the PLA segment, respectively, while the methylene protons (–CH_2_–) of the mPEG segment appear at 3.62 ppm. From the ratio of peak area at 5.14 and 3.62 ppm, the actual PLA-to-mPEG ratio after the followed ring-opening polymerization was 1/0.57.

Figure 7b shows the FT-IR spectra of the two neat monomers (PLA and mPEG), as well as the spectrum of the derived block copolymer. In regard to the neat PLA, several characteristic FT-IR peaks were recorded at 1740, 2992, 2941, and 1076 cm^−1^, corresponding to the stretching vibration of –C=O, asymmetric -CH_3_, symmetric –CH_3_ and C–O, respectively. Additionally, the bending frequencies of PLA corresponding to the asymmetric and symmetric –CH_3_ were recorded at 1448 and 1357 cm^−1^, respectively. In regard to neat mPEG, characteristic FT-IR peaks were recorded at approximately 3500 cm^−1^ (corresponding to the –OH vibrations), and several peaks in the region of 1500–1000 cm^−1^, with the more characteristic vibrations at 2850 and 2950 cm^−1^, corresponding to the -CH_2_ stretching of the mPEG. Looking at the obtained FT-IR spectrum of the mPEG-b-PLA, a strong absorption at 1760 cm^−1^ was recorded (which corresponds to the –C =O stretch of PLA), while the stretch of the C–O–C bands of the mPEG and PLA were also present at 1087 and 1184 cm^−1^, respectively. Additionally, the peaks of mPEG at ~3500 cm^−1^ (–OH vibration), 1087, and 1184 cm^−1^ (–CH_2_ stretching) were also seen. Hence, based on the obtained spectra, the ring-opening polymerization process followed was able to lead to the successful synthesis of the mPEG-b-PLA copolymer.

Following the above analyses, the thermal and physical state properties of the newly synthesized mPEG-b-PLA block copolymer were evaluated *via* DSC, TGA, and pXRD analysis.

**Figure 8:**
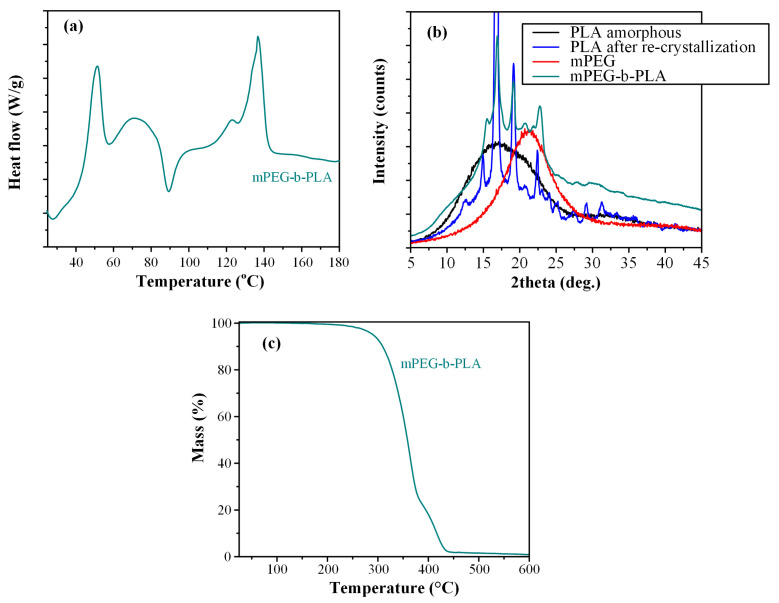
DSC thermogram (**a**), pXRD diffractograms (**b**), and TGA thermogram (**c**) of mPEG-b-PLA.

Figure 8a shows the DSC thermograms of the block copolymer. Results showed a first melting endotherm at 51.4 °C, attributed to the presence of the mPEG. Then, a small recrystallization exotherm was recorded at 89.5 °C, attributed to the PLA blocks, which is more readily crystallizable than the corresponding mPEG blocks, due to the higher inherent crystallization tendency of the lactic acid repeating units [53]. Finally, a broad melting endotherm was recorded at 136.7 °C, which is attributed to the presence of PLA [53]. Hence, based on the obtained DSC thermogram the prepared copolymer was semi-crystalline, showing the melting characteristics of both mPEG and PLA. However, since the *in situ* DSC amorphization or re-crystallization of the different monomers of the block-copolymer cannot be excluded, the physical state of the mPEG-b-PLA copolymer was further evaluated *via* pXRD.

Figure 8b shows the pXRD diffractograms for the neat mPEG and PLA monomers, as well as the newly synthesized mPEG-b-PLA block copolymer. The obtained diffractogram for the neat mPEG showed a broad pXRD peak at 21.3 deg, indicating that the monomer was semi-crystalline. In the case of neat PLA, pXRD diffractograms showed a characteristic amorphous halo, indicative of the amorphous nature of the polymer; however, upon melting and recrystallization, PLA showed several sharp diffractogram peaks at 15.2, 16.8, 19.1, and 22.4 deg, indicating that the polymer was recrystallized after processing. Looking now at the pXRD of the mPEG-b-PLA, results showed that the newly synthesized copolymer was semicrystalline, with several characteristic sharp diffractogram peaks, corresponding to the recrystallized PLA monomer.

Looking at the thermal stability properties of the prepared copolymer, the TGA thermogram recorded in Figure 8c showed that mPEG-b-PLA was thermally stable up to ~250 °C, indicating that the polyester is expected to be thermally stable during the preparation procedure of the thin-films, as well as during its storage (at least in normal storage conditions of pharmaceutical relevance, i.e., up to 40 °C). Finally, before proceeding with the preparation of thin-film patches, it should be noted that the intrinsic viscosity [η] of the prepared copolymer was estimated at 0.210 dL/g, while the same value for PLGA was 0.731 dL/g (both values may be considered as adequate, in relation to the concentrations selected later, for the preparation of polymeric solutions adequate for thin-film skin patches [54]).

### 3.4. LFD-Loaded NPs Embedded in Polyester Thin-Films

The purpose of the present study was to prepare a new LFD skin patch for the treatment of psoriasis. In this context, LDF-loaded NPs were prepared (using either neat or SDAEM-modified CS), in order to improve drug’s solubility and enhance the antibacterial properties of the resultant formulation. In this section, the use of two different polyesters (namely mPEG-b-PLA and PLGA) will be evaluated for the preparation of thin-films that will be able to successfully emend the previously prepared LFD-loaded NPs.

In the present study, the aforementioned films were prepared *via* spin-coating, following the process conditions described in the materials and methods section. In all cases (i.e., CS- or CS-SDAEM-based NPs with mPEG-b-PLA or PLGA), good polymeric thin-films were prepared with no agglomeration (or trapped air) and uniform structure. Table 3 summarizes the drug loading and EE values of the prepared LFD-NP-loaded polyester patches, with results showing good drug loadings and adequate EE in all cases.

**Figure 9:**
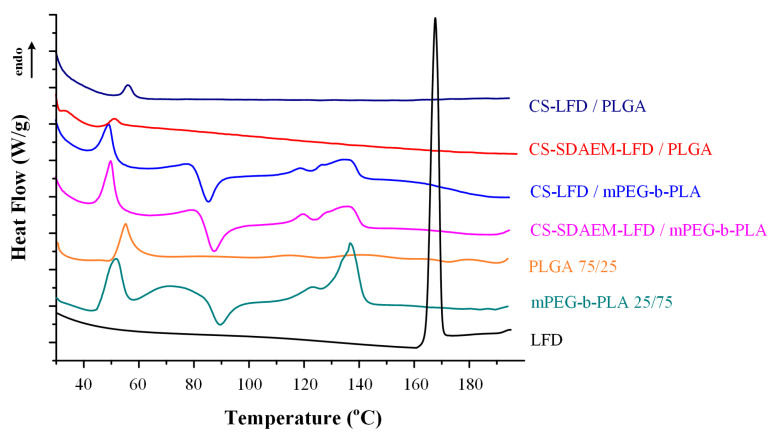
DSC thermogram of mPEG-b-PLA and PLGA thin-films with LFD-loaded NPs.

Changes in the physical state of the API and the polymeric matrices, during the preparation of the LFD-NP loaded thin-films, were initially evaluated with the aid of DSC (Figure 9). Results from the obtained thermograms showed that the API was amorphously dispersed, since no LFD melting endotherms were recorded, in all cases. Additionally, it should be pointed out that the thermal properties of the polymeric matrices were unaltered during the formation of the drug-loaded polymeric thin-films (as compared to the pure components).

**Figure 10:**
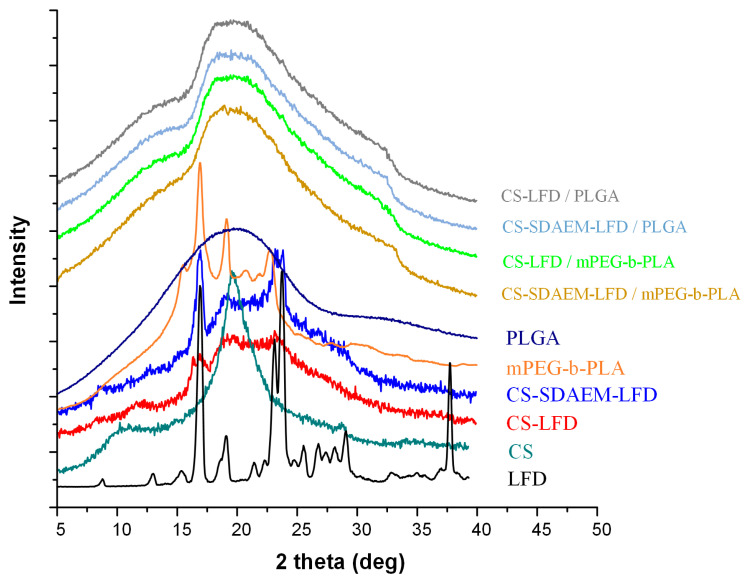
pXRD diffractograms of mPEG-b-PLA and PLGA thin-films with LFD-loaded NPs.

Since the *in situ* API amorphization during DSC cannot be excluded, and hence, the DSC suggestions of API’s amorphous dispersion within the prepared thin-films may be misleading, in addition to DSC, the physical state changes during the preparation of LFD-NP-loaded thin-films were also evaluated *via* pXRD (Figure 10).

Looking at the obtained diffractograms, it is obvious that all prepared drug-loaded thin-films were mainly amorphous, since a characteristic amorphous halo was observed in all recorded diffractograms. In addition, the recorded broad diffractogram peak at ~20 deg indicates that a small portion of the prepared films was crystalline (probably the polymeric part of mPEG-b-PLA or PLGA). Additionally, it should be noted that, based on obtained results, a significant decrease in mPEG-b-PLA’s crystallinity is seen after the preparation of the thin-films (as compared to the initially derived block copolymer). Similar results of amorphous matrix formation during thin-film preparation have been previously reported [55] and can be attributed to the spin-coating process followed.

**Figure 11:**
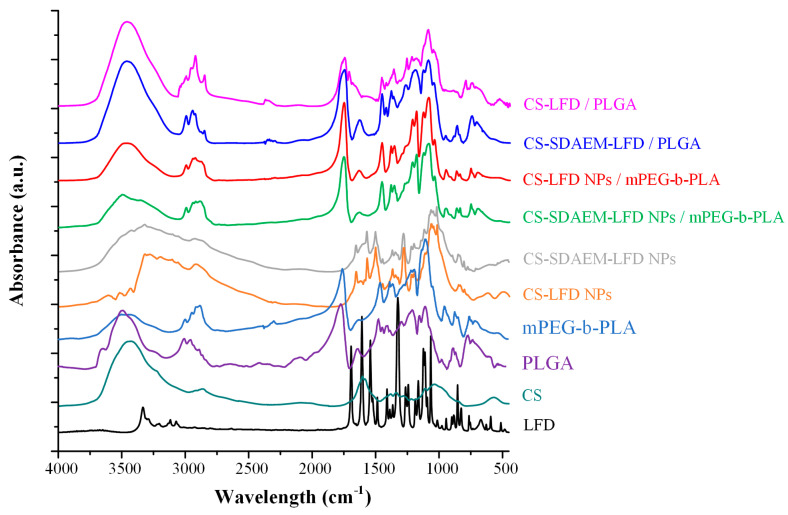
FT-IR spectra of mPEG-b-PLA and PLGA thin-films with LFD-loaded NPs.

In order to evaluate evolving molecular interactions between the drug-loaded NPs and the polymeric matrices, FT-IR spectroscopic analysis was conducted (Figure 11). Results in the case of PLGA-based thin-films showed that the obtained FT-IR spectra were the sum of the individual components (i.e., the spectra of the polymeric matrix and the drug-loaded NPs). This indicates that no molecular interactions (at least traceable by FT-IR analysis) were formed between the polymeric matrix (i.e., PLGA) and the embedded LFD-loaded NPs. On the contrary, the FT-IR spectra of the mPEG-b-PLA thin-films with SDAEM-modified CS showed a peak shift in the region of 3700–3000 cm^−1^, indicating thus that molecular interactions between the matrix carrier and the modified CS take place during the preparation of the drug-loaded thin-films (probably in the form of HBs).

**Figure 12:**
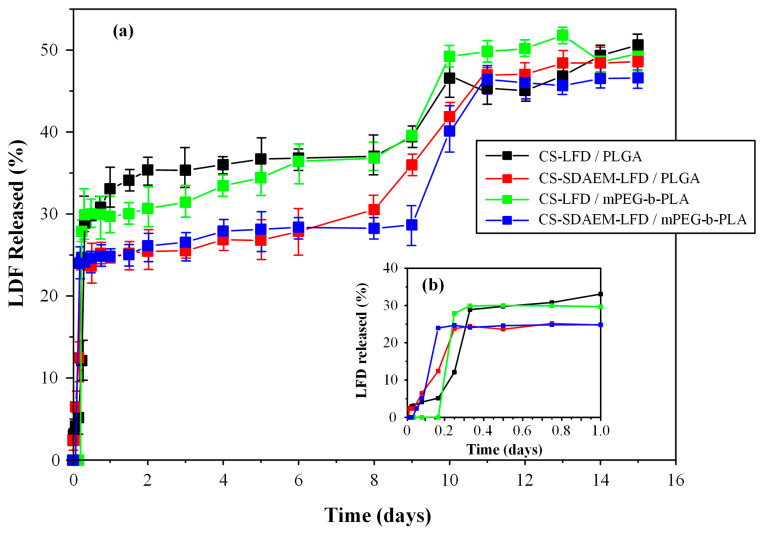
Full in vitro dissolution profile (**a**) and initial burst release (**b**) of mPEG-b-PLA and PLGA thin-films with LFD-loaded NPs.

Finally, Figure 12 depicts the in vitro dissolution profiles along with the initial burst release of the prepared drug-NP loaded thin-films.

Triphasic release profiles were observed for all examined formulations. Specifically, all obtained release profiles were characterized by: (i) an initial burst release phase until approximately 20 min (phase I), (ii) a steady drug prolong-releasing phase until ~8 days (phase II), and (iii) a faster 1^st^-order releasing phase until approximately 11 days (phase III). The initial burst release (i.e., phase I) can be attributed to the surface-located LFD-loaded NPs, while the steady drug prolonged-release phase and the following faster 1st-order-release phase are associated with the swelling (i.e., gel-forming ability) and the biodegradation (i.e., erosion) characteristics of the polyester matrices.

As can be observed in the given in vitro dissolution profiles, the formulations containing the unmodified CS displayed a more significant burst effect as compared to the LFD-NPs containing the SDAEM-modified CS. In addition, the latter released approximately 30% of the API in the first 0.3 days, and from then on, a sustained release profile reaching 35% of API in 8 days was recorded, while, at the same time, 27% and 25% of the API was released by CS-SDAEM NPs embedded in the PLGA or the mPEG-b-PLA polyester matrices, respectively. Taking into consideration the third API release phase, all formulations were able to release a good portion of the loaded API (~50%) within the first 11 to 12 days of the trial. Therefore, based on the obtained results, the drug-loaded CS-SDAEM NPs embedded in either polyester showed the most promising in vitro dissolution results, since an adequate extent of API was released in approximately ten days, while a reduced initial burst release was obtained. Comparison of the obtained dissolution results with similar, previously published, attempts using the active metabolite (teriflunomide) instead of LFD and poly(lactic acid)/poly(butylene adipate) blends for the preparation of electrospun nanofibers [13] shows that in the present study, the prepared films were able to sustain the API’s dissolution for a longer period of time (i.e., 12 as opposed to 5 days), although the previously prepared fiber mats were able to deliver a higher dose of the active metabolite.

It should be noted that the differences observed between the dissolution profiles in the case of the neat-drug-loaded NPs and the drug-loaded NPs embedded in the polyester films are due to the swelling and erosion (i.e., hydrolysis) rates of the polyesters used. Additionally, it can be assumed that during dissolution, a portion of the API is being diffused directly from the NPs into the swollen polyester matrices, and then being released by the two aforementioned mechanisms (swelling and erosion).

**Figure 13:**
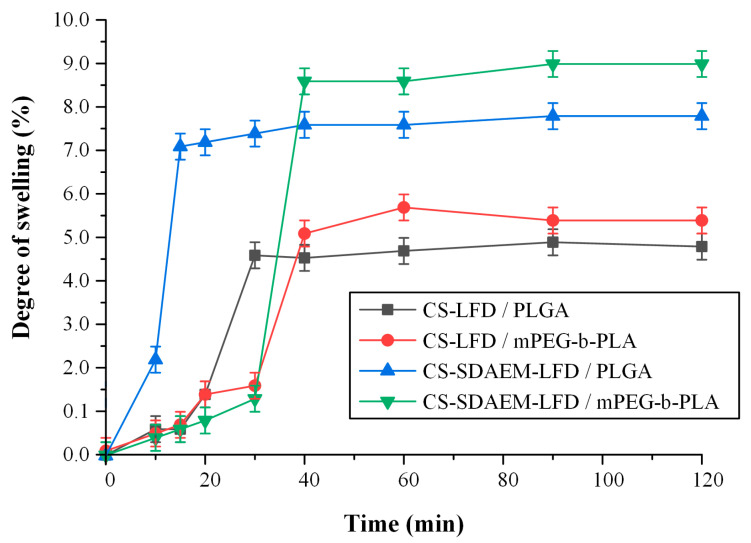
Degree of swelling vs. time of mPEG-b-PLA and PLGA thin-films with LFD-loaded NPs.

Finally, the swelling ratio of the prepared thin-films in SBF are presented in Figure 13. In general, the ability of a skin patch to absorb and retain water is crucial since it is considered to be one of the main mechanisms of regulating (sustaining) an API’s dissolution process, while increased swelling is related to increased cell adhesion and proliferation [56]. Moreover, in the case of psoriasis, retaining water is essential, since un-hydration in the site of action leads to losses in trans-epidermal water, which has shown to exacerbate the disease [57,58].

Results showed that in both cases (i.e., thin films prepared by PLGA and mPEG-b-PLA), the incorporation of drug-loaded NPs prepared *via* the SDAEM-modified CS resulted in a higher extent of swelling, and hence hydration, a feature that can be attributed to the slightly more hydrophilic nature of SDAEM-modified CS (already discussed in Section 3.1).

## 4. Conclusions

In the present study, drug-loaded NPs were successfully embedded in polymeric thin-films for the preparation of LFD-loaded skin patches. The use of SDAEM-grafted CS, even at low SDAEM to CS concentrations (i.e., 1 to ~16 w/w), resulted in the formation of drug-loaded NPs having better wetting properties and enhanced antibacterial characteristics compared to neat CS NPs. mPEG-b-PLA that was successfully synthesized *via* the ring-opening polymerization of L-lactide with mPEG as well as commercially available PLGA were used in the successful preparation of polymeric thin-films with embedded LFD-loaded CS NP. An in-depth evaluation of the prepared systems showed that the use of CS-SDAEM NPs (independent of the polyester type used for the preparation of films) may be considered as the most promising formulation approach and as a suitable solution for the preparation of an LFD-loaded thin-film skin patch.

## Figures and Tables

**Table 1:** NP’s DLS size and ζ-potential, as well as drug-loading, EE, and yield.

NPs	Particle Size (nm)	PDI	ζ- Potential (mV)	Drug-Loading (%)	ΕΕ (%)	Yield (%)
CS-LFD	371.06 ± 42	0.96	+57.7 ± 2.0	12.8 ± 1.0	47.9 ± 4.8	20.4 ± 3.4
CS-SDAEM-LFD	717.30 ± 23	0.58	+40.0 ± 1.3	8.2 ± 1.3	31.7 ± 3.6	20.0 ± 2.8
CS	352.67 ± 28	1.00	+59.9 ± 2.4	–	–	9.6 ± 1.5
CS-SDAEM	581.99 ± 35	0.38	+39.5 ± 1.8	–	–	15.3 ± 1.2

**Table 2:** Antibacterial activity of the pure CS, LFD, and the prepared NPs expressed in terms of colony-forming units (CFU) using *E. coli* and *S. aureus*.

Bacteria Type	Antibacterial Activity (CFU)					
	Control (Bacteria) *	Neat Components *			NPs *	
		CS	LFD	CS-SDAEM	CS-LFD	CS-SDAEM-LFD
*E. coli*	1.60 × 10^8^	1.20 × 10^8^	1.40 × 10^8^	0.95 × 10^8^	1.09 × 10^8^	0.08 × 10^8^
*S. aureus*	1.79 × 10^8^	1.50 × 10^8^	1.70 × 10^8^	1.30 × 10^8^	1.40 × 10^8^	1.12 × 10^8^

* All SD values were below 0.2.

**Table 3:** Drug loading and EE for the prepared LFD-loaded thin-films.

Sample—ID	Drug Loading (%)	EE (%)
*mPEG-b-PLA*		
CS-LFD	1.62 ± 0.05	72.04 ± 1.38
CS-SDAEM-LFD	1.58 ± 0.02	69.38 ± 0.97
*PLGA 25/75*		
CS-LFD	2.09 ± 0.09	77.29 ± 1.42
CS-SDAEM-LFD	1.73 ± 0.01	74.51 ± 1.07

## Data Availability

Data is contained within the article.

## References

[1] Sala M., Elaissari A., Fessi H. (2016). Advances in psoriasis physiopathology and treatments: Up to date of mechanistic insights and perspectives of novel therapies based on innovative skin drug delivery systems (ISDDS). J. Control Release.

[2] Mabuchi T., Chang T.W., Quinter S., Hwang S.T. (2012). Chemokine receptors in the pathogenesis and therapy of psoriasis. J. Dermatol. Sci..

[3] Pradhan M., Singh D., Singh M.R. (2013). Novel colloidal carriers for psoriasis: Current issues, mechanistic insight and novel delivery approaches. J. Control Release.

[4] Tang L., Yang X., Liang Y., Xie H., Dai Z., Zheng G. (2018). Transcription factor retinoid-related orphan receptor γt: A promising target for the treatment of psoriasis. Front. Immunol..

[5] Erol İ., Üstündağ Okur N., Orak D., Sipahi H., Aydın A., Özer Ö. (2020). Tazarotene-loaded in situ gels for potential management of psoriasis: Biocompatibility, anti-inflammatory and analgesic effect. Pharm. Dev. Technol..

[6] Pradhan M., Alexander A., Singh M.R., Singh D., Saraf S., Saraf S., Ajazuddin A. (2018). Understanding the prospective of nano-formulations towards the treatment of psoriasis. Biomed. Pharmacother..

[7] Armstrong A.W., Read C. (2020). Pathophysiology, clinical presentation, and treatment of psoriasis: A review. JAMA.

[8] Florek A.G., Wang C.J., Armstrong A.W. (2018). Treatment preferences and treatment satisfaction among psoriasis patients: A systematic review. Arch. Dermatol. Res..

[9] Padda I.S., Goyal A. (2020). Leflunomide. StatPearls.

[10] Boyd A.S. (2012). Leflunomide in dermatology. J. Am. Acad. Dermatol..

[11] Sehgal V.N., Verma P. (2013). Leflunomide: Dermatologic perspective. J. Dermatol. Treat..

[12] Prakash A., Jarvis B. (1999). Leflunomide: A review of its use in active rheumatoid arthritis. Drugs.

[13] Siafaka P.I., Barmbalexis P., Bikiaris D.N. (2016). Novel electrospun nanofibrous matrices prepared from poly(lactic acid)/poly(butylene adipate) blends for controlled release formulations of an anti-rheumatoid agent. Eur. J. Pharm. Sci..

[14] Xi H., Cun D., Xiang R., Guan Y., Zhang Y., Li Y., Fang L. (2013). Intra-articular drug delivery from an optimized topical patch containing teriflunomide and lornoxicam for rheumatoid arthritis treatment: Does the topical patch really enhance a local treatment?. J. Control. Release.

[15] Bae J., Park J.W. (2016). Topical delivery of leflunomide for rheumatoid arthritis treatment: Evaluation of local tissue deposition of teriflunomide and its anti-inflammatory effects in an arthritis rat model. Drug Dev. Ind. Pharm..

[16] Farokhzad O.C., Langer R. (2009). Impact of nanotechnology on drug delivery. ACS Nano.

[17] Rizeq B.R., Younes N.N., Rasool K., Nasrallah G.K. (2019). Synthesis, bioapplications, and toxicity evaluation of chitosan-based nanoparticles. Int. J. Mol. Sci..

[18] Zhang E., Xing R., Liu S., Qin Y., Li K., Li P. (2019). Advances in chitosan-based nanoparticles for oncotherapy. Carbohydr. Polym..

[19] Ahmed S., Ikram S. (2016). Chitosan based scaffolds and their applications in wound healing. Achiev. Life Sci..

[20] Jayakumar R., Prabaharan M., Sudheesh Kumar P.T., Nair S.V., Tamura H. (2011). Biomaterials based on chitin and chitosan in wound dressing applications. Biotechnol. Adv..

[21] Lin H.-T., Venault A., Chang Y. (2019). Zwitterionized chitosan based soft membranes for diabetic wound healing. J. Membr. Sci..

[22] Yilmaz Atay H. (2020). Antibacterial activity of chitosan-based systems. Funct. Chitosan.

[23] Rozman N.A.S., Tong W.Y., Leong C.R., Tan W.N., Hasanolbasori M.A., Abdullah S.Z. (2019). Potential antimicrobial applications of Chitosan Nanoparticles (ChNP). J. Microbiol. Biotechnol..

[24] Quan C., Chen X.Y., Li X., Xue F., Chen L.H., Liu N., Wang B., Wang L.Q., Wang X.P., Yang H. (2020). Psoriatic lesions are characterized by higher bacterial load and imbalance between Cutibacterium and Corynebacterium. J. Am. Acad. Dermatol..

[25] Jamaledin R., Yiu C.K.Y., Zare E.N., Niu L.-N., Vecchione R., Chen G., Gu Z., Tay F.R., Makvandi P. (2020). Advances in antimicrobial microneedle patches for combating infections. Adv. Mater..

[26] Mabrouk M., Kumar P., Choonara Y.E., Du Toit L.C., Pillay V. (2018). Artificial, triple-layered, nanomembranous wound patch for potential diabetic foot ulcer intervention. Materials.

[27] Carr L.R., Xue H., Jiang S. (2011). Functionalizable and nonfouling zwitterionic carboxybetaine hydrogels with a carboxybetaine dimethacrylate crosslinker. Biomaterials.

[28] Chang Y., Yandi W., Chen W.-Y., Shih Y.-J., Yang C.-C., Chang Y., Ling Q.-D., Higuchi A. (2010). Tunable bioadhesive copolymer hydrogels of thermoresponsive poly(N-isopropyl acrylamide) containing zwitterionic polysulfobetaine. Biomacromolecules.

[29] Mi L., Jiang S. (2014). Integrated antimicrobial and nonfouling zwitterionic polymers. Angew. Chem. Int. Ed..

[30] Liu G., Zhang L., Mao S., Rohani S., Ching C., Lu J. (2015). Zwitterionic chitosan–silica–PVA hybrid ultrafiltration membranes for protein separation. Sep. Purif. Technol..

[31] Chen Y., Li J., Li Q., Shen Y., Ge Z., Zhang W., Chen S. (2016). Enhanced water-solubility, antibacterial activity and biocompatibility upon introducing sulfobetaine and quaternary ammonium to chitosan. Carbohydr. Polym..

[32] Qi B., Kujawa P., Toita S., Beaune G., Winnik F.M. (2015). Phosphorylcholine-modified chitosan films as effective promoters of cell aggregation: Correlation between the films properties and cellular response. Macromol. Biosci..

[33] Cao Z., Wu M., Zhao Y., Dai L., Zeng R., Tu M., Zhao J. (2016). Bioinspired double-positively charged phosphodicholine-chitosan and zwitterionic phosphorylcholine-chitosan conjugates: The associated water structure, biocompatibility and antibacterial action. React. Funct. Polym..

[34] Sun N., Liu M., Wang J., Wang Z., Li X., Jiang B., Pei R. (2016). Chitosan nanofibers for specific capture and nondestructive release of CTCs assisted by pCBMA brushes. Small.

[35] Wang X., Tian Y., Zhao X., Peng S., Wu Q., Yan L. (2015). Effects of aeration position on organics, nitrogen and phosphorus removal in combined oxidation pond-constructed wetland systems. Bioresour. Technol..

[36] Ways T.M., Lau W.M., Khutoryanskiy V.V. (2018). Chitosan and its derivatives for application in mucoadhesive drug delivery systems. Polymers.

[37] Ma J., Zhong L., Peng X., Xu Y., Sun R. (2020). Functional chitosan-based materials for biological applications. Curr. Med. Chem..

[38] Wang W., Meng Q., Li Q., Liu J., Zhou M., Jin Z., Zhao K. (2020). Chitosan derivatives and their application in biomedicine. Int. J. Mol. Sci..

[39] Zhao D., Yu S., Sun B., Gao S., Guo S., Zhao K. (2018). Biomedical applications of chitosan and its derivative nanoparticles. Polymers.

[40] Bikiaris N.D., Michailidou G., Lazaridou M., Christodoulou E., Gounari E., Ofrydopoulou A., Lambropoulou D.A., Vergkizi-Nikolakaki S., Lykidou S., Nikolaidis N. (2020). Innovative skin product emulsions with enhanced antioxidant, antimicrobial and UV protection properties containing nanoparticles of pure and modified chitosan with encapsulated fresh pomegranate juice. Polymers.

[41] Yang S., Liu L., Han J., Tang Y. (2020). Encapsulating plant ingredients for dermocosmetic application: An updated review of delivery systems and characterization techniques. Int. J. Cosmet. Sci..

[42] Washington K.E., Kularatne R.N., Karmegam V., Biewer M.C., Stefan M.C. (2017). Recent advances in aliphatic polyesters for drug delivery applications. Wiley interdisciplinary reviews. Nanomed. Nanobio Technol..

[43] Wang M., Hu L., Xu C. (2017). Recent advances in the design of polymeric microneedles for transdermal drug delivery and biosensing. Lab Chip.

[44] Ita K. (2017). Dissolving microneedles for transdermal drug delivery: Advances and challenges. Biomed. Pharmacother..

[45] Xiao R.Z., Zeng Z.W., Zhou G.L., Wang J.J., Li F.Z., Wang A.M. (2010). Recent advances in PEG-PLA block copolymer nanoparticles. Int. J. Nanomed..

[46] Jain A.K., Goyal A.K., Gupta P.N., Khatri K., Mishra N., Mehta A., Mangal S., Vyas S.P. (2009). Synthesis, characterization and evaluation of novel triblock copolymer based nanoparticles for vaccine delivery against hepatitis B. J. Control. Release.

[47] Dong Y., Feng S.-S. (2006). Nanoparticles of poly(D,L-lactide)/methoxy poly(ethylene glycol)-poly(D,L-lactide) blends for controlled release of paclitaxel. J. Biomed. Mater. Res. Part A.

[48] Abdelrahman E.A., Abdel-Salam E.T., El Rayes S.M., Mohamed N.S. (2019). Facile synthesis of graft copolymers of maltodextrin and chitosan with 2-acrylamido−2-methyl−1-propanesulfonic acid for efficient removal of Ni(II), Fe(III), and Cd(II) ions from aqueous media. J. Polym. Res..

[49] Ardila N., Daigle F., Heuzey M.-C., Ajji A. (2017). Antibacterial activity of neat chitosan powder and flakes. Molecules.

[50] Solomon O.F., Ciutǎ I.Z. (1962). Détermination de la viscosité intrinsèque de solutions de polymères par une simple détermination de la viscosité. J. Appl. Polym. Sci..

[51] Karava A., Lazaridou M., Nanaki S., Michailidou G., Christodoulou E., Kostoglou M., Iatrou H., Bikiaris D.N. (2020). Chitosan derivatives with mucoadhesive and antimicrobial properties for simultaneous nanoencapsulation and extended cular release formulations of dexamethasone and chloramphenicol drugs. Pharmaceutics.

[52] Ing L.Y., Zin N.M., Sarwar A., Katas H. (2012). Antifungal activity of chitosan nanoparticles and correlation with their physical properties. Int. J. Biomater..

[53] Kyu S., Dukjoon C., Kim D. (2002). Drug-releasing behavior of MPEG/PLA block copolymer micelles and solid particles controlled by component block length. J. Appl. Polym. Sci..

[54] Pethrick R.A., Rankin K.E. (1999). Criteria for uniform thin film formation for polymeric materials. J. Mater. Sci..

[55] Nerantzaki M., Kehagias N., Francone A., Fernández A., Sotomayor Torres C.M., Papi R., Choli-Papadopoulou T., Bikiaris D.N. (2018). Design of a multifunctional nanoengineered PLLA surface by maximizing the synergies between biochemical and surface design bactericidal effects. ACS Omega.

[56] Terzopoulou Z., Michopoulou A., Palamidi A., Koliakou E., Bikiaris D. (2020). Preparation and evaluation of collagen-based patches as curcumin carriers. Polymers.

[57] Kim S.D., Huh C.H., Seo K.I., Suh D.H., Youn J.I. (2002). Evaluation of skin surface hydration in Korean psoriasis patients: A possible factor influencing psoriasis. Clin. Exp. Dermatol..

[58] Rim J.H., Jo S.J., Park J.Y., Park B.D., Youn J.I. (2005). Electrical measurement of moisturizing effect on skin hydration and barrier function in psoriasis patients. Clin. Exp. Dermatol..

